# Meta-analysis of grain yield QTL identified during agricultural drought in grasses showed consensus

**DOI:** 10.1186/1471-2164-12-319

**Published:** 2011-06-16

**Authors:** BP Mallikarjuna Swamy, Prashant Vikram, Shalabh Dixit, HU Ahmed, Arvind Kumar

**Affiliations:** 1 International Rice Research Institute, DAPO Box 7777, Metro Manila, Philippines

## Abstract

**Background:**

In the last few years, efforts have been made to identify large effect QTL for grain yield under drought in rice. However, identification of most precise and consistent QTL across the environments and genetics backgrounds is essential for their successful use in Marker-assisted Selection. In this study, an attempt was made to locate consistent QTL regions associated with yield increase under drought by applying a genome-wide QTL meta-analysis approach.

**Results:**

The integration of 15 maps resulted in a consensus map with 531 markers and a total map length of 1821 cM. Fifty-three yield QTL reported in 15 studies were projected on a consensus map and meta-analysis was performed. Fourteen meta-QTL were obtained on seven chromosomes. MQTL_1.2_, MQTL_1.3_, MQTL_1.4_, and MQTL_12.1 _were around 700 kb and corresponded to a reasonably small genetic distance of 1.8 to 5 cM and they are suitable for use in marker-assisted selection (MAS). The meta-QTL for grain yield under drought coincided with at least one of the meta-QTL identified for root and leaf morphology traits under drought in earlier reports. Validation of major-effect QTL on a panel of random drought-tolerant lines revealed the presence of at least one major QTL in each line. *DTY*_*12.1 *_was present in 85% of the lines, followed by *DTY*_*4.1 *_in 79% and *DTY*_*1.1 *_in 64% of the lines. Comparative genomics of meta-QTL with other cereals revealed that the homologous regions of MQTL_1.4 _and MQTL_3.2 _had QTL for grain yield under drought in maize, wheat, and barley respectively. The genes in the meta-QTL regions were analyzed by a comparative genomics approach and candidate genes were deduced for grain yield under drought. Three groups of genes such as stress-inducible genes, growth and development-related genes, and sugar transport-related genes were found in clusters in most of the meta-QTL.

**Conclusions:**

Meta-QTL with small genetic and physical intervals could be useful in Marker-assisted selection individually and in combinations. Validation and comparative genomics of the major-effect QTL confirmed their consistency within and across the species. The shortlisted candidate genes can be cloned to unravel the molecular mechanism regulating grain yield under drought.

## Background

Drought is a severe abiotic stress that affects the production and productivity of rice. Drought stress at the reproductive stage is the most devastating [1,2]. Because of the ongoing process of climate change, the rainfall pattern has become more irregular in the cropping season, causing widespread drought in rice-growing areas, which results in severe yield losses [3,4]. The development of drought-tolerant varieties that maintain good yield under drought is a priority area of rice research for sustainable rice production.

Marker-assisted mapping and introgression of major-effect QTL for grain yield under drought could be an efficient and fast-track approach for breeding drought-tolerant rice varieties [5]. However, the successful use of QTL in marker-assisted selection depends on their effect and consistency across genetic backgrounds and environments. Most of the QTL for grain yield under drought have been mapped against a single genetic background in early-segregating generations (F_3_, BC_2_, and BC_2_F_2_) evaluated in a limited number of environments. Such QTL may not provide a consistent effect because of variation in the genetic background and environment. Additionally, the QTL may not be transferrable to other backgrounds because of unfavorable epistatic interactions resulting in reduced or even no effects in a new genetic background [6,7]. Considering all these facts, it is difficult to predict the usefulness of QTL for MAS based only on their performance in an individual genetic background in any particular study.

A more efficient way to select QTL for MAS is to compare the identified QTL with earlier reported studies for their consistency of location and effect across genetic backgrounds and environments. Consistently identified QTL at the same chromosomal location, explaining high phenotypic variance and having a major effect on a trait, can be effectively used in MAS [8-10].

QTL meta-analysis is an approach to identify consensus QTL across studies, to validate QTL effects across environments/genetic backgrounds, and also to refine QTL positions on the consensus map [11]. QTL meta-analysis requires independent QTL for the same trait obtained from different populations, different locations, or different environmental conditions [11]. The consistent QTL identified by meta-analysis for a set of QTL at a confidence interval (CI) of 95% are called meta-QTL (MQTL). The meta-QTL with the smallest CI and having a consistent and large effect on a trait are useful in MAS. In plants, the concept of meta-analysis has been applied to the analysis of QTL/genes for blast resistance [12], root traits and drought tolerance in rice [9,10], lint fiber length in cotton [13], cyst nematode resistance in soybean [14], fusarium head blight resistance in wheat [15], flowering time [16], drought tolerance in maize [17], and disease resistance in cocoa [18].

QTL validation is another approach to confirm the effect of QTL across different genetic backgrounds. QTL regions harbor many genes; among them, a few key genes could be more important in the regulation of a complex trait. Meta-QTL regions with refined positions are more accurate for short-listing of candidate genes. The common candidate genes short-listed across the meta-QTL are more likely candidates that regulate yield [9].

In this study, QTL meta-analysis was carried out for yield QTL under drought to develop a consensus map and to identify consensus yield QTL under drought with the objective to provide markers of MQTL with high effects and small confidence intervals for possible use in MAS or for fine-mapping QTL for gene discovery. Also, markers linked to 12 major QTL for grain yield were validated on a set of random drought-tolerant lines, including landraces and improved drought breeding lines developed at IRRI, to know the frequency of their universal presence. Further, a comparative genomics approach was used to identify the homologous regions of MQTL in other cereal crops such as maize, sorghum, wheat, and barley (http://www.gramene.org/,http://www.maizegdb.org/, http://www.graingenes.org).

## Materials and methods

### Meta-QTL analysis

Three steps were employed for the identification of a consensus QTL for grain yield under drought. First, in a bibliographic review, reliable data on QTL for yield per plant were compiled. Second, a consensus map was created and on this map the QTL of individual studies was projected. In the third step, a meta-analysis was performed on QTL clusters to identify the consensus MQTL.

### Bibliographic review and synthesis of yield QTL data

QTL information was collected from published reports involving mapping of QTL for grain yield under drought. There were 15 reports of a QTL mapping for grain yield under drought. The details of the parents used in developing the mapping population, size of the mapping population, markers used, and yield QTL identified are given in Table 1. In all, 53 QTL were reported for yield.

**Table 1:** Details of mapping studies undertaken for grain yield under drought QTL

S. no.	Parents used in crossing	Mapping population	Population size	Number of markers	Markers used	Number of locations used for phenotyping	Yield QTL identified	References
1	CTM9993-5-10-1 × IR62266-42-6-2	DH	154	280	AFLP, RFLP, SSR	2	4	[37]
2	CTM9993-5-10-1 × IR62266-42-6-2	DH	154	315	AFLP, RFLP, SSR	1	7	[1]
3	Zhenshan 97 × IRAT109	RIL	180	245	SSR	2	4	[38]
4	Zhenshan 97B × IRAT109	RIL	187	213	SSR	2	2	[39]
5	Zhenshan 97 × IRAT109	RIL	180	245	SSR	2	5	[40]
6	IR20 × Nootripathu	RIL	150	51	SSR	1	2	[22]
7	Bala × Azucena	RIL	177	163	SSR, AFLP, RFLP, BAC markers	1	4	[41]
8	CTM9993-5-10-1 × IR62266-42-6-2	DH	220	315	AFLP, RFLP, SSR	3	1	[42]
9	Vandana × Way Rarem	F_3:4, _BC_2_	436	126	SSR	2	3	[5]
10	Apo × Swarna	BC_1_, BC_2_, BC_3_	301	293(BSA) 13(WG)	SSR	2	4	[2]
11	N22 × Swarna	F_3:4_	292	140(BSA) 17(WG)	SSR	2	4	[23]
12	N22 × MTU1010	F_3:4_	362	140(BSA) 125	SSR	2	5	[23]
13	N22 × IR64	F_3:4_	289	140(BSA) 13(WG)	SSR	2	4	[23]
14	IR77298-14-1-2 × IR64	BC_1_, BC_2_, BC_3_	288	18	SSR	3	3	IRRI, Unpublished
15	IR55419-04 × Way Rarem	RIL	158	3	SSR	2	1	IRRI, Unpublished

BSA = bulk segregant analysis; WG = whole genotyping; AFLP = amplified fragment length polymorphism; RFLP =restricted fragment length polymorphism, SSR = simple sequence repeats, BAC = bacterial artificial chromosome

### Development of a consensus map

A consensus genetic map was constructed and meta-analysis was performed using Biomercator v2.0 (http://www.genoplante.com/). The rice genetic linkage map of Temnykh et al. [19] was used as a reference map, on which the markers of 15 studies were projected to develop a consensus map. Chromosomes connected with fewer than two common markers to the reference map were excluded before the creation of the consensus map. Inversions of marker sequences were filtered out by discarding inconsistent loci with the exception of very closely linked markers. After the integration of all maps, the consensus map contained 531 markers, including SSR, RFLP, AFLP markers, and genes. The consensus map covered a total length of 1821 cM, with an average distance of 3.5 cM between markers.

### QTL projections

For all studies, the 95% confidence intervals of initial QTL on their original maps were estimated using the approach described by Darvasi and Soller [20]:

Where N is the population size and R^2 ^the proportion of the phenotypic variance explained by the QTL. The CI was re-estimated to control the heterogeneity of CI calculation methods across studies. Projection of QTL positions was performed by using a simple scaling rule between the original QTL flanking marker interval and the corresponding interval on the consensus chromosome. For a given QTL position, the new CI on the consensus linkage group was approximated with a Gaussian distribution around the most likely QTL position. All projections of QTL onto the consensus map were performed using the Biomercator (2.0) (http://www.genoplante.com/).

### Meta-analysis

Meta-analysis was performed on the QTL clusters on each chromosome using Biomercator (2.0) (http://www.genoplante.com). The Akaike Information Criterion (AIC) was used to select the QTL model on each chromosome [21]. According to this, the QTL model with the lowest AIC value is considered a significant model indicating the number of meta-QTL. QTL meta-analysis requires independent QTL for the same trait obtained from different plant populations, different locations, or different environmental conditions [11].

### QTL validation

#### Genotyping

All molecular marker work was conducted in the Gene Array and Molecular Marker Analysis (GAMMA) Laboratory, Plant Breeding, Genetics and Biotechnology (PBGB) division, IRRI. For DNA extraction, freeze-dried samples were used. Freeze-dried leaf samples were cut in eppendorf tubes and ground through a GENO grinder. Extraction was carried out by the modified CTAB method. DNA samples were stored in 2-mL deep-well plates (Axygen Scientific, California, USA). DNA samples were quantified on 0.8% agarose gel and concentration adjusted to approximately 25 ng μL^-1^. PCR amplification was done with a 15-μL reaction mixture having 40 ng DNA, 1 × PCR buffer, 100 μM dNTPs, 250 μM primers, and 1 unit *Taq *polymerase enzyme. The PCR profiles started with an initial denaturation of DNA at 94°C for 5 minutes, followed by 35 amplification cycles of denaturation at 94°C for 1 minute, annealing temperatures varied from 55°C to 58°C for 45 seconds based on the primer, extension at 72°C for 1 minute and final extension at 72°C for 7 minutes. The PCR products were resolved on 8% non-denaturing polyacrilamide gels (PAGE). The gels were scored taking respective QTL donor alleles as reference band and scores were used for QTL validation. The details of the peak markers of the 12 major effect QTL are given in Additional File 1.

Twelve major effect drought grain yield QTL were validated on a panel of 92 drought tolerant lines consisting of traditional drought tolerant donors, drought tolerant breeding lines developed through conventional breeding approaches and random high yielding lines under drought from QTL mapping populations. The peak marker of all the twelve major effect QTL were amplified on the drought panel lines. The lines were scored taking QTL donor allele as a base. The list of lines is given in the Additional File 2.

### Gene content analysis

The 14 meta-QTL were analyzed for gene content to know the presence of genes and gene clusters responsible for drought. A comparative genomics approach was followed to analyze the genes present in meta-QTL. Gene content was noted based on annotated data of homologous regions in Nipponbare using RAP, Build5 (http://rapdb.dna.affrc.go.jp/download/index.html). It is assumed that the genes identified in Nipponbare regions are homologous and collinear to those underlying the yield QTL under drought mapped in different studies involving different donors and recipients.

### Comparative genomics to identify homologous regions in cereals

A comparative genomics approach was followed to identify homologous regions between rice and maize using the genomic databases (http://www.gramene.org). Homologous regions identified were checked for the presence of drought grain yield QTL of maize (http://www.maizegdb.org). In sorghum, wheat, and barley, grain yield QTL reported were collected from a literature survey and these were compared with the meta-QTL using the comparative maps available in the Gramene database (http://www.gramene.org).

## Results and discussion

### Overview of QTL and development of a consensus map

**Figure 1:**
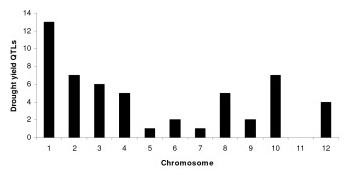
**Distribution of grain yield QTL on rice chromosomes**. The bar diagram depicts the frequency of drought grain yield QTLs on rice chromosomes

In the 15 populations of rice screened for drought tolerance to map QTL, population size ranged from 150 [22] to 436 lines [5]. The number of markers used ranged from 13 to 315 [1,23]. The number of locations for phenotyping varied from 1 to 3. From the 15 studies, 53 yield QTL were reported, which were distributed on all the chromosomes except chromosome 11 (Table 1). The number of QTL per population ranged from 1 to 7. The proportion of QTL per chromosome ranged from one QTL each on chromosomes 5 and 7 to 18 yield QTL on chromosome 1. The distribution of yield QTL on different chromosomes showed that chromosomes 1, 2, and 10 have the highest number, 18, 7, and 7 QTL, respectively (Figure 1). The phenotypic variance of the initial QTL varied from 3.2% to 40% and the confidence interval of the markers varied from 2 to 30 cM. The rice genetic map of Temnykh et al. [19] was used as a reference map to develop a consensus map as this is a widely used genetic map of rice and it contained most of the markers used in the different studies. The consensus map consisted of 531 markers with a total map length of 1821 cM. The average distance between the markers was 3.5 cM, thus enabling the identification of a precise location of QTL. There were very few marker inversions in the consensus map, which were discarded from the final map and further analysis.

### Meta-analysis and QTL validation

**Figure 2:**
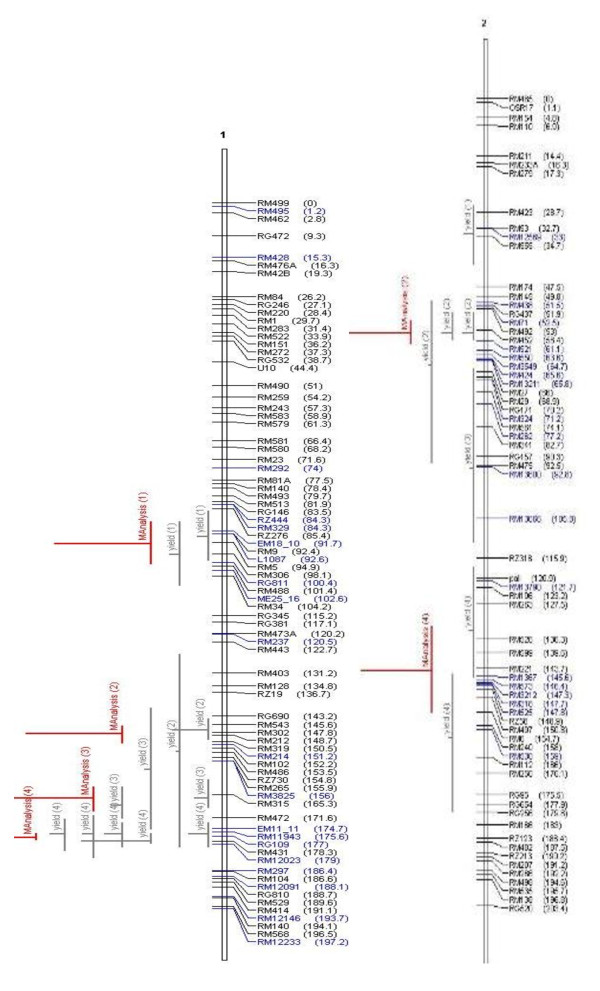
**Meta-QTLs identified on chromosomes 1 and 2 by Meta- analysis of reported yield QTLs**. The picture shows the Meta-QTLs on chromosomes 1 and 2. Vertical lines on the left of chromosomes indicate the confidence interval, horizontal lines indicate the variance, MQTL are in red. Markers and genetic distance (cM) are shown on the right of chromosomes.

**Figure 3:**
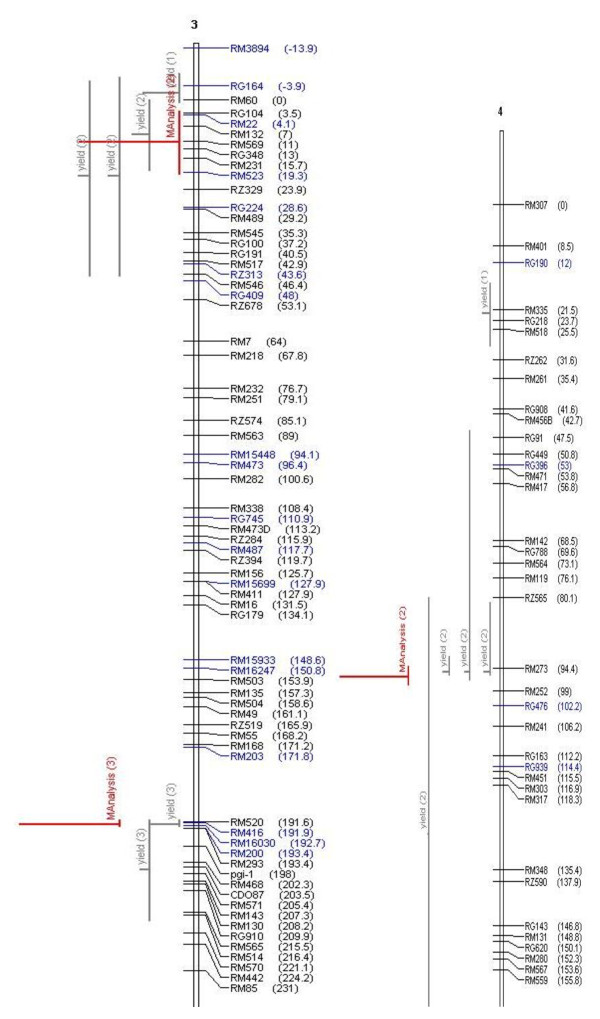
**Meta-QTLs identified on chromosomes 3 and 4 by Meta- analysis of reported yield QTLs**. The picture shows the Meta-QTLs on chromosomes 3 and 4. Vertical lines on the left of chromosomes indicate the confidence interval, horizontal lines indicate the variance, MQTL are in red. Markers and genetic distance (cM) are shown on the right of chromosomes.

**Figure 4:**
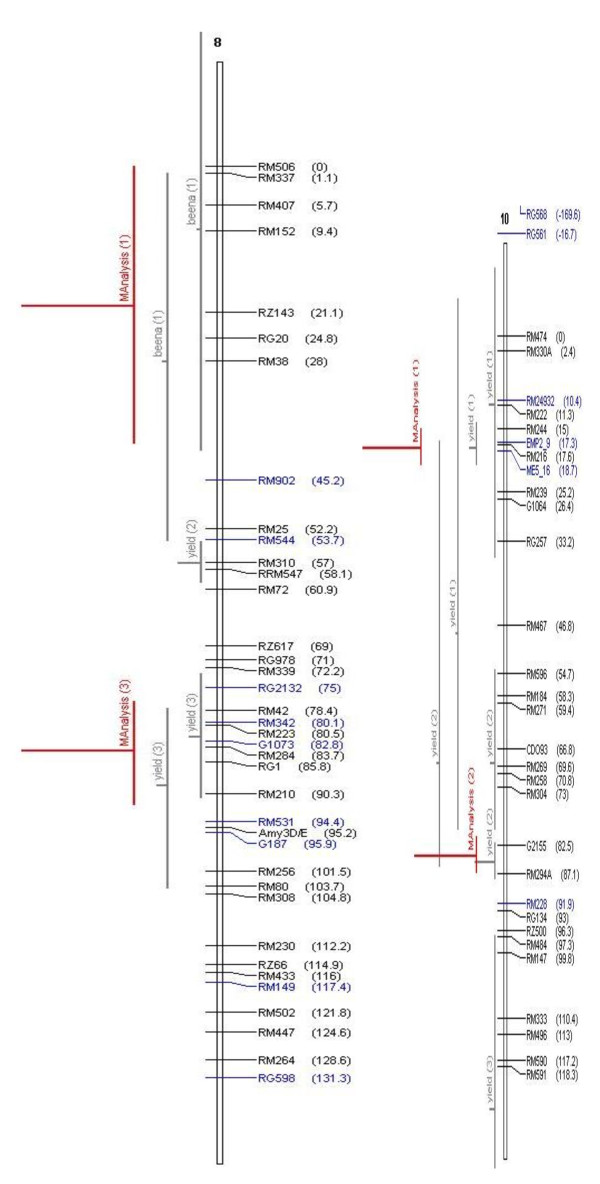
**Meta-QTLs identified on chromosomes 8 and 10 by Meta- analysis of reported yield QTLs**. The picture shows the Meta-QTLs on chromosomes 8 and 10. Vertical lines on the left of chromosomes indicate the confidence interval, horizontal lines indicate the variance, MQTL are in red. Markers and genetic distance (cM) are shown on the right of chromosomes.

**Figure 5:**
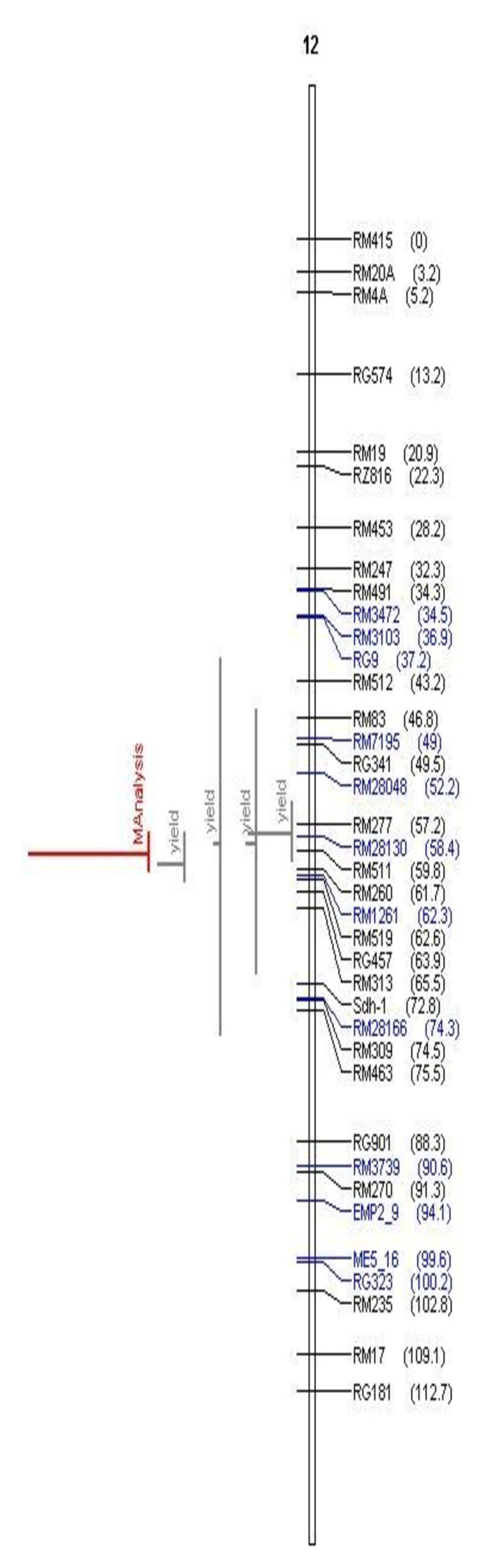
**Meta-QTLs identified on chromosome 12 by Meta- analysis of reported yield QTLs**. The picture shows the Meta-QTLs on chromosome 12. Vertical lines on the left of chromosomes indicate the confidence interval, horizontal lines indicate the variance, MQTL are in red. Markers and genetic distance (cM) are shown on the right of chromosomes.

**Figure 6:**
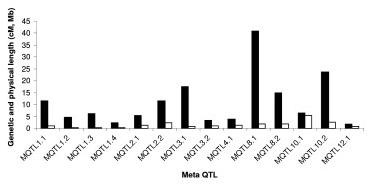
**Genetic and physical intervals of MQTL**. The diagram depicts the genetic and physical intervals of the MQTLs. Solid bars indicates genetic interval (cM) and hollow bars indicates the physical interval (Mb) of the Meta-QTL.

**Figure 7:**
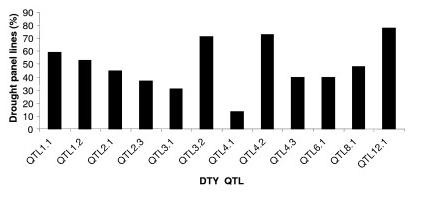
**Frequency of drought grain yield QTL in drought panel lines**. The diagram depicts frequency of major effect drought grain yield QTLs in a drought panel consisting of 92 lines.

It is widely believed that QTL are accurate and can be positioned onto chromosomal locations by molecular mapping [24,25]. However, their complex nature and context dependency in different genetic backgrounds and environments are constraints in identifying their precise location. The identification of the most accurate and precise major-effect QTL across genetic backgrounds and environments is a prerequisite for the successful use of QTL in MAS. Meta-analysis of QTL identified in different studies helps to identify the most precise and concise QTL, which can be further pursued for MAS or the identification of candidate genes. In our study, we attempted to identify the meta-QTL for grain yield under drought by genome-wide meta-analysis. From a bibliographic survey, a total of 53 QTL were short-listed for grain yield under drought from 15 studies. All 53 QTL were projected on a consensus map. The chromosomal regions with only one QTL were not considered for meta-analysis since meta-analysis by definition involves more than one QTL. Thus, 38 QTL were used for meta-analysis and meta-QTL were short-listed based on the Akaike Information Criterion (AIC). Accordingly, the QTL model with the lowest AIC value was considered a significant model indicating the number of meta-QTL. The number of meta-QTL along with their AIC values and confidence intervals are given in Table 2. In total, 14 independent meta-QTL were identified at a confidence interval of 95% on seven chromosomes, and meta-analysis successfully reduced the total QTL by 63% (Figures. 2, 3, 4, 5). The meta-QTL identified on each chromosome varied from 1 to 4. There were four meta-QTL on chromosome 1; two on chromosomes 2, 3, 8 and 10; and one each on chromosomes 4 and 12. The phenotypic variance of the meta-QTL varied from 4% to 28%. At 10 of the 14 meta-QTL, the mean phenotypic variance was more than 10%. In general, the confidence intervals at most of the meta-QTL were narrower than their respective original QTL. At nine loci on chromosomes 1, 2, 3, 4, 10, and 12, meta-QTL were narrower than the mean of their initial QTL. However, at five loci, the meta-QTL were broader than the mean of the initial QTL. The confidence intervals of the meta-QTL varied from 2.4 cM between the marker intervals RG109 and RM431 on chromosome 1 to 40.8 cM between the marker intervals RM337 and RM902 on chromosome 8. At two regions, meta-QTL_1.4 _(MQTL_1.4) _and MQTL_12.1_, the CI declined to around 2 cM. The physical intervals of the meta-QTL varied from 0.16 Mb to 5.3 Mb. Three meta-QTL were less than 500 kb. The meta-QTL regions with small genetic and physical intervals are useful in MAS. It is significant to see that seven QTL that had less than 1.3 Mb intervals also had a genetic interval of around 6 cM with a phenotypic variance of more than 10% (Figure 6). Three of these meta-QTL were on chromosome 1 and one each on chromosomes 2, 3 and 12. The physical intervals of MQTL_1.2_, MQTL_1.3_, and MQTL_1.4 _were less than 400 kb, that of MQTL_12.1 _was 700 kb, and those of MQTL_3.2_, MQTL_4.1_, and MQTL_2.1 _were 1Mb, 1.3 Mb, and 1.2 Mb, respectively. MQTL_1.2_, MQTL_3.2_, and MQTL_12.1 _had phenotypic variance of more than 20%. The seven MQTL regions with small genetic and physical intervals are important regions for MAS, fine mapping, candidate gene identification, and functional analysis. These QTL can be introgressed in popular rice mega-varieties to develop drought-tolerant and high-yielding lines. In addition to meta-analysis of QTL, the markers linked to the 12 major-effect QTL for grain yield were also validated on a panel of drought-tolerant lines to confirm their presence in larger set of lines. It is notable that major-effect QTL *DTY*_*12.1 *_was present in 85% of the lines. *DTY*_*3.2*_, *DTY*_*4.2 *_*DTY*_*1.1*_, *DTY*_*8.1*_, and *DTY*_*1.2 *_were present in more than 50% of the lines (Figure 7). The amplification of the RM523 and RM11943 peak markers of *DTY*_*3.2 *_and *DTY*_*1.1 *_in a set of 92 drought tolerant panel lines is presented in Additional File 3. The result indicates the presence of at least one of the major-effect grain yield QTL in the drought panel lines. In general, the major-effect QTL identified for grain yield under drought have a genetic gain of 10% to 30%, with a yield advantage of around 150 to 500 kg/ha over recipient parents. However, considering practical benefit to farmers, the development of drought-tolerant rice varieties with a yield advantage of at least 1 ton/ha could be the desired target for rice breeders. The marker-aided QTL pyramiding of the major-effect MQTL identified in this study can be considered as an option for achieving this target.

**Table 2:** Meta-QTL for yield under drought identified by meta-analysis

S. no.	MQTL	Chromosome	QTL region	AIC value	QTL model	No of initial QTL	Mean phenotypic variance of the QTL	Mean initial CI (cM)	MQTL CI (95%) (cM)	Physical length of MQTL (Mb)	kb/cM	Coefficient of reduction in length from mean initial QTL to MQTL	MQTL rank for MAS/fine mapping
1	MQTL_1.1_	1	RZ276-RM488	146.2	4	2	16	7.50	11.50	1.14	103.1	0.7	
2	MQTL_1.2_	1	RM543-RM212			3	24	5.20	4.53	0.27	60.3	1.1	2
3	MQTL_1.3_	1	RM315-RM472			2	16	17.80	6.30	0.16	183.4	2.8	4
4	MQTL_1.4_	1	RG109-RM431			5	12	7.60	2.40	0.36	151.5	3.2	5
5	MQTL_2.1_	2	RM452-RM521	62.7	4	3	12	10.50	5.28	1.24	229.8	2.0	
6	MQTL_2.2_	2	RM526-RM497			2	6	12.00	11.50	2.36	110.7	1.0	
7	MQTL_3.1_	3	RG104-RM523	45.2	3	3	13	5.40	17.43	0.84	47.7	0.3	
8	MQTL_3.2_	3	RM520-RM16030			2	20	10.30	3.40	0.98	488.0	16.6	3
9	MQTL_4.1_	4	RM273-RM252	45.3	3	3	9	8.40	3.98	1.32	338.2	2.1	
10	MQTL_8.1_	8	RM337-RM902	40.4	3	2	4	4.00	40.87	1.90	48.0	0.0	
11	MQTL_8.2_	8	RM339-RM210			2	15	7.50	14.95	1.90	132.0	0.5	
12	MQTL_10.1_	10	RM244-ME5_16	61.8	4	2	4	13.00	6.50	5.30	825.0	2.0	
13	MQTL_10.2_	10	RM596-RM304			3	16	15.00	23.72	2.60	112.0	0.6	
14	MQTL_12.1_	12	RM277-RM260	21.2	1	4	28	4.20	1.79	0.70	178.3	2.3	1

AIC = Akaike Information Criterion, CI = confidence interval, cM = centiMorgan, MQTL = meta-QTL; kb = kilobase, MB = megabase

A comparison was made between the meta-QTL identified in this study with the meta-QTL identified for root traits in two earlier studies [9,10]. It is very interesting to note that MQTL_1.2_, MQTL_2.2_, MQTL_3.1_, MQTL_4.1_, and MQTL_8.2 _coincided with QTL clusters for root and leaf morphology traits associated with drought tolerance/avoidance in rice [9]. All the 14 independent meta-QTL coincided with at least one meta-QTL identified for root traits under drought [10]. Earlier studies on meta-analysis of QTL for root traits [9,10] and blast resistance in rice [12], fusarium head blight resistance in wheat [15], flowering time in maize [16], nematode resistance in soybean [14], and lint fiber length in cotton [13] identified precise and concise meta-QTL. Meta-QTL were also used to deduce candidate genes and were recommended for MAS in some of these studies.

### Comparative genomics of MQTL

**Figure 8:**
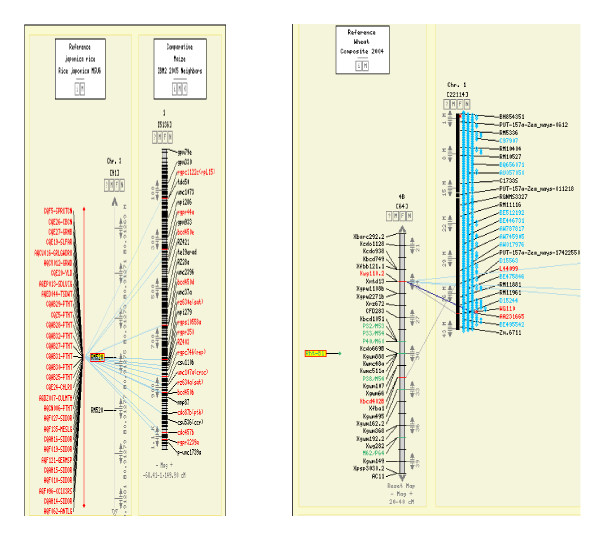
**Comparative map of MQTL**_**1.4 **_**in rice with its corresponding grain yield QTL near *Rht-b1 *in wheat**. The picture shows the comparative location of major effect Meta-QTL for grain yield under drought in rice MQTL_1.4 _on a wheat genetic map.

The existence of an evolutionary relationship among the grass families is a well-known fact. The syntenic relationship can be used to identify the homologous regions among these species, which in turn is useful in defining their role in plant growth, development, and adaptation across species. We compared meta-QTL regions for synteny in other cereal crops. The major-effect MQTL_1.4 _was also found in maize on chromosome 3 near marker *msu2*, in wheat on chromosome 4B near marker *Rht-b1*, and in barley on chromosome 6H near marker *Bmac0316*, while major-effect MQTL_3.2 _was also found in maize on chromosome 1 near marker *Umc107a *(Figure 8). All these markers were linked to grain yield under drought in their respective crops. The largest parts of chromosomes 1 and 3 of rice have a syntenic relation with chromosomes 3 and 1 of maize, so their respective homologous QTL were also found on the corresponding chromosomes. An interesting observation is that, near the *sd1 *locus on chromosome 1 of rice, QTL for grain yield under drought were identified most frequently. *Sd1 *is a major locus responsible for semidwarf plant stature in rice and its corresponding locus in wheat is *Rht-b1 *on chromosome 4B. MQTL_1.4 _is near the *sd1 *locus and also on its corresponding locus *Rht-b1 *in wheat, major QTL for grain yield under drought were detected.

### Gene content analysis and identification of candidate genes

Meta QTL with precise and narrow confidence intervals are useful in short listing the candidate genes. Using the annotated gene information available in the rice database, the genes present in the 14 meta-QTL regions were analyzed by comparative genomics approach and candidate genes were shortlisted. The short-listed candidate genes can be further confirmed by transgenic approaches by loss or gain of function studies. Most of the genes present in the MQTL were genes for hypothetical and expressed proteins, pseudo genes, genes for signal transduction, and transposable elements. However, there were many annotated genes/gene families that were common across the MQTL regions; these are probable candidate genes for yield under drought. It was found that three kinds of genes frequently occurred together in these regions. The genes/gene families were stress-inducible genes, growth and development-related genes, and sugar transport-related genes. Table 3 lists the important genes underlying MQTL for grain yield under drought. In six MQTL with less than a 1 Mb region, LRR kinase, leucine zipper, cell division-controlling proteins, sugar transport protein-like genes, no apical meristem (*NAM*), pentatricopeptide repeat proteins, cytokinin oxidase, F-box proteins, AP2-domain containing proteins, and zinc-finger transcription factors were present. The candidacy of these genes for yield and yield traits has already been proved in rice and other crops. Cytochrome P450 has a role in bassinosteroid homeostasis and had an influence on leaf angle leading to increased yield in rice [26,27]. Pentatricopeptide repeats are present in the promoter region of *Rf *genes, which restore fertility and also play a role in embryogenesis in *Arabidopsis *[28,29]. Zinc-finger (AN1-like)-like proteins are known to be involved in stress tolerance. Zinc-finger protein in rice are induced after different types of stresses, namely, cold, desiccation, salt, submergence, heavy metals, and mechanical injury. Over expression of the zinc-finger gene in transgenic tobacco conferred tolerance of cold, dehydration, and salt stress at the seed germination/seedling stage [30,31]. F-box proteins play an important role in floral development and stress tolerance. In addition, F-box proteins appear to serve as the key components of the machinery involved in regulating plant growth and development throughout the plant's life cycle and their expression is influenced by light and abiotic stresses [32]. Leucine zippers are a class of transcription factor involved in ABA-independent stress tolerance. Over expression of *OsbZIP23 *in rice triggered clusters of genes regulating stress adaptations [33]. The no apical meristem gene (*NAM*) plays an important role in the growth and development of meristematic tissue. The root-specific expression of this gene resulted in enhanced root growth and improved drought tolerance in rice [34]. The other important genes that harbored the meta-QTL were the *ERECTA *and *DREB *genes. *ERECTA *is a leucine-rich repeat receptor-like kinase gene known for its influence on inflorescence development, stomatal density, epidermal cell expansion, and mesophyll cell proliferation. This gene is mainly involved in transpiration efficiency and enhanced drought response [35]. *DREB *is a well-known transcription factor that is induced by drought and it activates many down stream stress-responsive genes to ultimately improve the drought and chilling tolerance of rice [36]. Some of these short-listed genes can be considered as positional candidate genes that determine grain yield under drought. However, it is also well known that yield and adaptability to stress are complex in nature and highly negatively correlated. The QTL/genes for these two are often co-located. Even though individual genes have been proved to regulate yield under controlled drought experiments, a well-coordinated response of many genes is essential for drought tolerance under field conditions. This is evident from the presence of three different groups of gene clusters in most of the meta-QTL regions.

**Table 3:** Candidate genes reported in the identified MQTL region.

S. no.	MQTL	Candidate genes (no. within MQTL)	Candidate genes	Candidate genes (no. in total)
1	MQTL_1.1_	1	Calcineurin-related phosphoesterase-like	14
2		2	ERECTA-like kinase 1-like	35
3		3	Putative ankyrin-kinase	69
4		4	Putative NAC transcription factor	135
5		5	Putative pectin acetylesterase precursor	139
6		6	Putative signal recognition particle	160
7		7	QUAKING isoform 5-like	179
8		8	Tetratricopeptide repeat (TPR)-containing protein-like	193
9	MQTL_1.2_	1	ABC transporter subunit-like	1
10		2	F-box domain-containing protein-like	39
11		3	Glutaredoxin-like	43
12		4	Leucine zipper protein-like	51
13		5	Lustrin A-like	52
14		6	Nodulin-like protein	57
15		7	Ovate family protein-like	59
16		8	Pentatricopeptide repeat (PPR)-containing protein-like	60
17		9	Protein kinase-like	66
18		10	Putative auxin-independent growth promoter	76
19	MQTL_1.3_	1	Cell wall protein-like	21
20		2	Cytochrome P450 monooxygenase	30
21		3	F-box domain-containing protein-like	39
22		4	hAT dimerisation domain-containing protein-like	45
23		5	HGWP repeat-containing protein-like	48
24		6	Leucine zipper protein-like	51
25		7	Nucleoporin-like protein	58
26		8	Pentatricopeptide repeat (PPR)-containing protein-like	60
27		9	pr1-like protein	65
28		10	Sucrose-phosphatase-like protein	192
29		11	Zinc knuckle domain-containing protein-like	206
30	MQTL_1.4_	1	Polyprotein-like	64
31		2	Putative aspartic proteinase nepenthesin II	74
32		3	Putative cytokinin oxidase	97
33		4	Putative lectin-like receptor kinase 1:1	130
34		5	Putative vacuole membrane protein 1	172
35	MQTL_2.1_	1	Ethylene-responsive family protein-like	37
36		2	Putative cytochrome P450	94
37		3	Putative DREPP2 protein	106
38		4	Putative F-box protein	111
39		5	Putative flavin-containing monooxygenase	114
40		6	Putative GTP-binding protein	120
41		7	Putative kaurene synthase	128
42		8	Putative pentatricopeptide repeat (PPR)-containing protein	140
43		9	Putative sugar transporter	164
44		10	Aquaporin	7
45	MQTL_2.2_	1	Cell wall protein	21
46		2	Dehydration-responsive family protein-like	33
47		3	F-box protein-like	39
48		4	Growth-regulating factor 1-like	44
49		5	HGWP repeat-containing protein-like	48
50		6	Pentatricopeptide repeat (PPR)-containing protein-like	60
51		7	Putative anther-specific protein	70
52		8	Putative anthocyanin biosynthetic gene regulator	72
53		9	Putative basic-helix-loop-helix transcription factor	77
54		10	Putative cell division control protein	85
55		11	Putative cold acclimation protein	90
56		12	Putative CRT/DRE binding factor 1	93
57		13	Putative cytochrome P450	94
58		14	Putative growth-regulating factor 1	119
59		15	Putative high-mobility group protein	124
60		16	Putative pectin methylesterase	138
61		17	Putative photoperiod-independent early flowering	145
62		18	Putative sexual differentiation process protein	158
63		19	Root-specific protein	184
64		20	Sexual differentiation process protein-like	187
65		21	Trehalose-6-phosphate phosphatase	194
66		22	UDP-glycosyltransferase-like	194
67		23	Vesicle-associated membrane protein-like	199
68		24	Zinc finger (C3HC4-type RING finger)-like	201
69	MQTL_3.1_	1	Adapitin protein-like	4
70		2	Cell division control protein 2-like	18
71		3	Cyclin 2 interactor-like	26
72		4	F-box domain-containing protein-like	39
73		5	Flavanone 3-hydroxylase-like	40
74		6	HGWP repeat-containing protein-like	48
75		7	MADS-box transcription factor	53
76		8	NAC domain-containing protein-like	54
77		9	Photomorphogenic	63
78		10	Putative callose synthase 1	81
79		11	Putative cell cycle switch protein	84
80		12	Putative cell division control protein 2	86
81		13	Putative cytochrome p450	94
82		14	Putative dihydrodipicolinate reductase	103
83		15	Putative dihydrofolate synthetase	104
84	MQTL_3.2_	1	ABC transporter-like protein-like	2
85		2	c-type cytochrome synthesis 1	25
86		3	Pentatricopeptide repeat (PPR)-containing protein-like	60
87		4	Pherophorin-dz1 protein-like	62
88		5	Putative cleavage stimulation factor subunit 1-like protein	89
89		6	Putative cold acclimation protein	90
90		7	Putative peroxidase	142
91		8	Putative phytochrome C	146
92		9	Putative prolamin	148
93		10	Putative prolyl 4-hydroxylase	149
94		11	Putative protein kinase SPK-2	150
95		12	Putative protein phosphatase 2C	151
96		13	Putative UDP-glucose 6-dehydrogenase	171
97		14	Putative zinc-finger protein	177
98		15	Receptor protein kinase	181
99		16	Senescence downregulated leo1	185
100	MQTL_4.1_	1	Auxin-related protein-like	11
101		2	Cell division cycle	20
102		3	Cytochrome c oxidase	28
103		4	Hydroxyproline-rich glycoprotein	49
104		5	Integral membrane transporter-like	50
105		6	Lustrin A-like	52
106		7	Protoporphyrinogen IX oxidase	67
107		8	Putative calcium-binding protein	79
108		9	Putative cell cycle checkpoint protein MAD2 homolog	83
109		10	Putative chitinase	88
110		11	Putative CONSTANS-like protein	92
111		12	Putative ER33 protein	108
112		13	Putative LRR receptor-like kinase	131
113		14	Putative salt-tolerance protein	154
114		15	Stress-inducible protein	191
115		16	Zinc finger (C3HC4-type RING finger)-like protein	202
116	MQTL_4.2_	1	ABC-1-like	3
117		2	Auxin response factor	9
118		3	Calcium-dependent protein kinase	15
119		4	CCAAT-box binding factor HAP5	17
120		5	Cytochrome P450 monooxygenase	29
121		6	Cytokinin-induced apoptosis inhibitor 1	32
122		7	Heat shock protein binding	47
123		8	HGWP repeat-containing protein	48
124		9	Pentatricopeptide repeat (PPR)-containing protein	60
125		10	Pherophorin-C1 protein precursor-like	61
126		11	Putative calcium-dependent protein kinase	80
127		12	Putative dehydration-responsive element-binding protein	101
128		13	Putative ethylene response factor	109
129		14	Putative floricaula	115
130		15	Putative flowering locus D	116
131		16	Putative growth-regulating factor	118
132		17	Putative IAA24	125
133		18	Putative inositol 1,3,4,5,6-pentakisphosphate 2-kinase	126
134		19	Putative jasmonate O-methyltransferase	127
135		20	Putative late embryogenesis abundant protein	129
136		21	Putative wall-associated kinase 1	175
137		22	RCP1 (ROOT CAP 1)-like	180
138		23	Stress-related-like protein interactor-like	190
139		24	Wall-associated protein kinase-like	200
140		25	Zinc finger (C3HC4-type RING finger)-like	201
141	MQTL_8.1_	1	Heat shock protein	46
142		2	Vesicle-associated membrane protein	197
143		3	Auxin efflux carrier protein-like	8
144		4	Cell division control protein-like	19
145		5	Cellulose synthase-1-like protein	22
146		6	CLAVATA1 receptor kinase (CLV1)-like protein	23
147		7	CONSTANS-like protein	24
148		8	Cytochrome b5-like	27
149		9	Ethylene-responsive elongation factor EF-Ts precursor-like	36
150		10	F-box domain-containing protein-like	39
151		11	Germin protein type 1	42
152		12	HGWP repeat-containing protein-like	49
153		13	NAC2 protein-like	55
154		14	Nam-like protein	56
155		15	Nodulin-like protein	57
156		16	Pentatricopeptide repeat (PPR)-containing protein-like	60
157		17	Polyprotein-like protein	64
158		18	Putative ABC transporter	68
159		19	Putative anthocyanin 5-aromatic acyltransferase	71
160		20	Putative AP2/EREBP transcription factor LEAFY PETIOLE	73
161		21	Putative CCAAT box binding factor/transcription factor Hap2a	82
162		22	Putative chaperone GrpE	87
163		23	Putative cold shock protein-1	91
164		24	Putative cytokinin-regulated kinase 1	98
165		25	Putative death receptor interacting protein	99
166		26	Putative DEFECTIVE IN ANTHER DEHISCENCE1	100
167		27	Putative farnesylated protein	110
168		28	Putative fertility restorer homolog	113
169		29	Putative MADS-box protein	132
170		30	Putative male fertility protein	133
171		31	Putative nucleoporin	137
172		32	Putative pherophorin	143
173		33	Putative senescence-associated protein	155
174		34	Putative osmatic embryogenesis receptor-like kinase 1	178
175		35	Putative sexual differentiation process protein isp4	159
176		36	Putative starch synthase	161
177		37	Putative stress-responsive gene	162
178		38	Putative teosinte branched1 protein	166
179		39	Putative trehalose-6-phosphate synthase	170
180		40	Putative vesicle-associated membrane associated protein	173
181		41	Putative wall-associated kinase	174
182		42	Ripening-related protein-like	182
183		43	Root cap protein 1-like	183
184		44	Senescence-associated protein-like	186
185		45	Stress-inducible protein-like	189
186		46	Zinc finger-like protein	204
187	MQTL_8.2_	1	AP2 domain transcription factor-like	6
188		2	Auxin-induced protein-related-like protein	10
189		3	F-box protein family-like protein	39
190		4	Pentatricopeptide repeat (PPR)-containing protein-like	60
191		5	Putative calcineurin B subunit	78
192		6	Putative cytochrome P450 monooxygenase	95
193		7	Putative male fertility protein	133
194		8	Putative NAC domain protein	134
195		9	Putative senescence-associated protein	155
196		10	Putative stromal cell-derived factor 2 precursor	163
197		11	Putative temperature stress-induced lipocalin	165
198		12	Putative teosinte branched1 protein	166
199		13	Putative tethering factor	167
200		14	Putative trehalose-6-phosphate synthase	170
201		15	Somatic embryogenesis receptor kinase-like protein	188
202		16	Zinc finger protein-like	204
203	MQTL_10.1_	1	Aminotransferase-like	5
204		2	Putative gibberellin-regulated protein	117
205		3	Putative peptide transporter 1	141
206		4	Putative serine threonine kinase	157
207		5	Putative wall-associated kinase 4	176
208		6	ABC transporter-like	1
209	MQTL_10.2_	1	Calcineurin B-like protein	13
210		2	Calcium-dependent protein kinase, isoform 1 (CDPK 1)	16
211		3	Cytochrome p450-like	31
212		4	Dehydration-responsive family protein-like	33
213		5	Elicitor-like protein	34
214		6	Ethylene-responsive protein-like	38
215		7	F-box protein-like	39
216		8	Fringe-related protein-like	41
217		9	Pentatricopeptide repeat (PPR)-containing protein-like	60
218		10	Putative anther-specific protein	70
219		11	Putative auxin response factor 10	75
220		12	Putative cytokinin dehydrogenase	96
221		13	Putative DEFECTIVE IN ANTHER DEHISCENCE1	100
222		14	Putative dehydration-induced protein	102
223		15	Putative DRE binding factor 2	105
224		16	Putative drought-inducible protein	107
225		17	Putative fertility restorer	112
226		18	Putative hairy meristem	121
227		19	Putative heat shock factor RHSF5	122
228		20	Putative hexose carrier protein HEX6	123
229		21	Putative *NAM *(no apical meristem) gene	136
230		22	Putative pollen-specific kinase partner protein	147
231		23	Putative root cap-specific glycine-rich protein	152
232		24	Putative salt-induced MAP kinase 1	153
233		25	Putative senescence-associated protein DH	156
234		26	Putative tonoplast membrane integral protein	168
235		27	Putative trehalose-6-phosphate phosphatase	169
236		28	Putative zinc finger protein	177
237		29	Ripening-related protein-like	182
238		30	Senescence-associated protein-like	186
239		31	Stress-inducible protein-like	189
240		32	Tetratricopeptide repeat (TPR)-containing protein-like	193
241		33	Universal stress protein-like	195
242	.	34	Vacuolar protein-sorting 13C protein-like	196
243		35	Vesicle-associated membrane associated protein-like	198
244		36	Zinc finger (HIT type)-like	203
245	MQTL_12.1_	1	Calcineurin B-like	12
246		2	Cell wall protein-like	21
247		3	HGWP repeat-containing protein-like	48
248		4	Hydroxyproline-rich glycoprotein-like	49
249		5	Putative pherophorin-dz1 protein	144
250		6	Zinc knuckle-containing protein-like	205

## Conclusions

Meta-analysis of grain yield QTL is an effective approach in identifying concise and precise consensus QTL. The seven meta-QTL identified with small genetic and physical intervals could be useful in MAS/pyramiding. Validation of the major-effect QTL confirmed the consistency of the major-effect grain yield QTL under drought in different drought-tolerant panel lines. The comparative genomics approach to identify the consistency of drought grain yield QTL across species revealed the conservation of some of the loci, indicating their evolutionary significance. The presence of gene clusters in the meta-QTL indicates that a well-coordinated response of many genes is essential to achieve drought tolerance under field conditions.

## Authors' contributions

AK conceived the idea of Meta- analysis, QTL validation and comparative genomics of grain yield QTL under drought. BPMS compiled and analyzed the data, carried out the QTL validation and comparative genomics. SD helped in data compilation and analysis. BPMS, PV, SD, HUA and AK were responsible for drafting and editing the manuscript. All authors have read and approved the final manuscript.

## Supplementary Material

Additional File 1 **Details of the markers used for QTL validation**. This file contains the list of major effect QTLs for grain yield under drought and peak markers of the QTLs. Primer sequence, product size of the markers and annealing temperatures (Tm) used for amplifying the markers. Click here for file

Additional File 2 **Drought panel lines for QTL validation**. This table shows the list of drought panel lines and type of the breeding material. These lines were used for validating the major effect QTLs for grain yield under drought. Click here for file

Additional File 3 **Amplification of RM523 and RM11943 peak markers of *QTL***_***3.2 ***_**and *QTL***_***1.1 ***_**in a set of 92 drought tolerant panel lines**. The gel picture shows the amplication of RM523 and RM11943 peak markers of *QTL*_*3.2 *_and *QTL*_*1.1 *_in a set of 92 drought tolerant panel lines. Click here for file
