# Supplementary material for: Meta-analysis of grain yield QTL identified during agricultural drought in grasses showed consensus
Source: BMC Genomics. 2011 Jun 16;12:319. doi: 10.1186/1471-2164-12-319 (PMC3155843; doi:10.1186/1471-2164-12-319)
Supplement: Additional File 1 — Details of the markers used for QTL validation. This file contains the list of major effect QTLs for grain yield under drought and peak markers of the QTLs. Primer sequence, product size of the markers and annealing temperatures (Tm) used for amplifying the markers. [file 1471-2164-12-319-S1.DOC]

Additional File 1: Details of the markers used for QTL validation

| S.No | DTY QTL | Marker | Forward Pimer | Reverse Primer | Product Size (bp) | Tm(^o^C) |
| --- | --- | --- | --- | --- | --- | --- |
| 1 | QTL_1.1_ | RM11943 | CTTGTTCGAGGACGAAGATAGGG | CCAGTTTACCAGGGTCGAAACC | 77 | 57 |
| 2 | QTL_1.2_ | RM6703 | GCTTTCCTCTCCTCTCCTCTCC | CAAATCAGTGTCGTATGCAGTGG | 200 | 55 |
| 3 | QTL_2.1_ | RM324 | GATTCCACGTCAGGATCTTCTGG | GCTCACCAGTTGAGATTGAAAGG | 196 | 55 |
| 4 | QTL_2.3_ | RM573 | TCATGTTGACGCACACATACACG | CTCTTCTTCCCTGGACCACACC | 144 | 56 |
| 5 | QTL_3.1_ | RM520 | ACGATAACGCCGACATCACTGG | GCTAAGCATCCACGGTTTCTCTCC | 114 | 57 |
| 6 | QTL_3.2_ | RM523 | TGAATTCTTGCACATGGTCAGC | TGGGAGGTTTGCTAGGGTAATCC | 252 | 58 |
| 7 | QTL_4.1_ | RM17435 | GCCAATGTGTGAGAAGAGGATAGG | GAGGCAAGCTTTCTACCATTATGC | 369 | 57 |
| 8 | QTL_4.2_ | RM131 | GGAGCAGCTTCTCGAGCATGG | CCAAATCTCGCCTCGTTTAGCC | 258 | 56 |
| 9 | QTL_4.3_ | RM518 | AAGACACAAGCAAACAGCTCAACC | AAGCTTGCTTGGTTCAAGAGAGG | 193 | 58 |
| 10 | QTL_6.1_ | RM510 | GTTTGACGCGATAAACCGACAGC | ATGAGGACGACGAGCAGATTCC | 193 | 57 |
| 11 | QTL_8.1_ | RM256 | GACAGGGAGTGATTGAAGGC | GTTGATTTCGCCAAGGGC | 105 | 55 |
| 12 | QTL_12.1_ | RM511 | AACGAAAGCGAAGCTGTCTCC | ATTTGTTCCCTTCCTTCGATCC | 143 | 55 |
